# Psychological Health Risk Factors Among Healthcare Workers: A Structural Equation Modeling Approach

**DOI:** 10.3390/healthcare14152280

**Published:** 2026-07-27

**Authors:** Zemiao Zhang, Yanna Li, Baoxiang Song, Kui Wang, Feng Hu

**Affiliations:** 1 School of Health Economics and Management, Nanjing University of Chinese Medicine, Nanjing 210023, China; 2 Shenzhen Health Development Research and Data Management Center, Shenzhen 518048, China; 3 College of Economics and Management, Nanjing Forestry University, Nanjing 210037, China; 4 Institute of International Business and Economics Innovation and Governance, Shanghai University of International Business and Economics, Shanghai 201620, China

**Keywords:** healthcare workers, psychological health, risk formation, structural equation model

## Abstract

**Background:** The psychological health of healthcare workers is a critical global public health concern. Identifying associated risk factors and their interrelationships is key to informing the development of effective interventions. This study aimed to construct a risk factor model for psychological health among healthcare workers and to examine the strength and patterns of associations between various factors and psychological health outcomes using structural equation modeling. **Methods:** A cross-sectional survey was conducted among 930 healthcare professionals. Structural equation modelling (SEM) was used to test the theoretical hypotheses, with bootstrapping used for path significance and multiple indicators multiple causes (MIMIC) models used for robustness validation. **Results:** The social environment and job demand risks were greater than the negative events and psychological adjustment risks. The final model explained 62.2% of the variance in psychological health. The social environment and organizational management functioned as distal risk factors, exerting indirect effects. Job demands, negative events, and job resource risks were associated with psychological health through psychological adjustment. Most of the hypothesized direct associations with the outcomes–burnout, anxiety, depression, suicidal ideation, and turnover intentions—were significant, except for the direct effects of job resource risk on anxiety and job demand/resource risk on suicidal ideation. MIMIC analysis confirmed the stability of the model. **Conclusions:** The proposed model clarifies how multilevel risk factors interact to affect healthcare workers’ psychological health. The findings support the development of comprehensive, prioritized interventions, including strengthened legal protections, dynamic staffing schemes in high-pressure departments, and routine psychological resilience training for staff.

## 1. Introduction

Medical practice is characterized by high professionalism and social responsibility, with healthcare workers facing unique occupational challenges. While healthcare professionals are well equipped with medical knowledge to maintain health, the demanding and stressful nature of their work frequently results in detrimental health outcomes. COVID-19 has highlighted the incidence of psychological distress among medical staff. A study from Wuhan revealed that frontline healthcare workers experienced depression (50.4%), anxiety (44.6%), insomnia (34.0%), and pain (71.5%) [1]. Another systematic review that included 38 studies revealed that healthcare workers with posttraumatic stress disorder, anxiety, depression, and pain accounted for 49%, 40%, 37%, and 37%, respectively [2]. After the pandemic, the problem has persisted. Sevliya et al. reported that under the normalization of the pandemic, up to 49.4%, 54.8%, and 33.3% of healthcare workers still experience anxiety, depression, and stress, respectively [3]. Maxudova et al. found that approximately two-thirds of clinical nurses suffer from occupational burnout [4]. Wang et al. demonstrated that medical staff members exhibit significantly higher sleep disorder scores than nonmedical populations do, with strong correlations with anxiety and depression [5].

The primary burden of psychological health disorders lies not in treatment costs but in the depletion of human capital and diminished productivity. Prolonged emotional distress among healthcare workers undermines both job satisfaction and clinical decision-making, thus degrading the quality of care. Recognizing this, global authorities have increasingly framed staff well-being as a cornerstone of health system resilience. The theme of World Patient Safety Day in 2020 was *Healthcare Safety: The Top Priority for Achieving Patient Safety*. The *World Mental Health Report* released by the WHO emphasizes that healthcare workers are a high-risk group for psychological problems and urgently need increased attention and systematic support from all sectors of society [6].

Previous studies have typically used psychological health as the dependent variable and employed regression analysis or other methods to examine the risk factors affecting the psychological health of medical staff. Elbay et al. reported that compared with male healthcare workers, female healthcare workers experience higher levels of stress, anxiety, and depression, potentially because of hormonal influences [7]. Data from Asian countries indicate that psychological issues are most prevalent among staff in the respiratory, emergency, ICU, and infectious disease departments [8]. According to an online survey conducted by the British Medical Association, as doctors work longer hours, they are more likely to suffer from psychological and emotional disorders [9]. During the COVID-19 pandemic, owing to the lack of pandemic prevention materials, fear of infection became a prominent risk factor affecting the psychological status of medical staff [10]. Wang et al. reported that 67.5% of hospital violence victims, including victims of emotional abuse and physical aggression, exhibited depressive symptoms [11]. Additionally, better perceived doctor–patient relationships and stronger social support are correlated with fewer psychological problems [12]. Some medical staff, viewing their profession as overly sacred, suppress negative emotions or avoid seeking help to protect the industry’s reputation, thereby exacerbating occupational burnout [13]. Although most healthcare workers maintain a positive attitude, unaddressed negative emotions may escalate into severe mental health issues.

Risk formation refers to the combination and interaction among risk-inducing factors, ultimately leading to adverse consequences [14,15]. Unlike physical illnesses, mental health risks are more insidious and less studied than chronic disease risks are, with existing research emphasizing risk factor interactions. Song et al. used a logistic regression model to construct a psychological health risk assessment model for police officers, including their duty mode, police category, emotional response, physical response, etc. [16]. Similarly, Xiao et al. proposed a model for submarine crews, evaluating environmental adaptation, occupational recognition, interpersonal relationships, life satisfaction, work pressure, health concerns, and self-regulation [17]. Other scholars have applied factor analysis and structural equation modelling (SEM) to analyse how environmental attributes, neighbourhood relationships, and perceived risks affect pregnant women’s psychological health [18]. Ribeiro-Gonçalves et al. further employed SEM to explain the mechanisms linking loneliness, ageing, and psychological resilience to mental health risks among elderly people [19].

The aforementioned findings provide valuable references for this study. However, existing research has primarily examined isolated factors, resulting in fragmented findings with inconsistent conclusions regarding risk factor typologies and effect sizes. Current studies moreover tend to adopt a microlevel individual perspective, despite the unique occupational context of healthcare workers, where psychological issues span individual, professional, organizational, and societal dimensions. This incomplete characterization of psychological risk factors and their mechanisms hinders both a comprehensive understanding of the occupational well-being of medical staff and the development of targeted interventions.

To address these gaps, this study aims to identify and model the interrelationships among psychological health risk factors among healthcare professionals using SEM and robustness validation. The remainder of the paper is structured as follows. Section 2 develops an integrated theoretical framework. Section 3 details the methodology for validating this framework. Section 4 presents the empirical results, focusing on the structural relationships and the strength of associations among the risk factors. Section 5 provides an interpretation of the findings, compares them with the literature, and discusses the implications. Section 6 concludes the paper by summarizing its key contributions.

## 2. Research Model Development

### 2.1. Theoretical Foundations

(1) Epidemiological triangle model

This theory suggests that the core elements of disease occurrence include the host, agent, and environment [20]. The term “host” refers to an organism that is susceptible to infection by a pathogen and whose specific characteristics may influence its susceptibility to the disease. The term “agent” refers to factors that can cause a certain disease and are often the direct cause of the disease. The term “environment” refers to external conditions. Under normal circumstances, the three elements maintain a dynamic balance, and the individual is in a healthy state. Once one or more factors change and exceed the threshold, the balance is disrupted, leading to the occurrence of disease. The epidemiological triangle model was initially proposed to explain the occurrence of infectious diseases, but with changes in the human disease spectrum and health needs, it can still provide valuable perspectives for explaining noncommunicable diseases and injury events [20]. Its theoretical value lies in the proposal of an analytical framework for disease dynamics, which helps researchers identify risk factors that affect health. Given that the triangle model has been used to study issues such as fatigue [21], panic [22], suicide [20], and occupational health [23], this study suggests that it is suitable for identifying psychological health risk factors among healthcare workers.

(2) Job demands–resources (JD-R) model

The JD-R model posits that occupational strain arises from an imbalance between high job demands and limited job resources [24]. Job demands encompass factors reflecting the quantitative and qualitative workload, including emotional labour, interpersonal stressors, and task overload, which require sustained physical and psychological effort from employees. Conversely, job resources comprise organizational and interpersonal factors that facilitate goal attainment, mitigate exhaustion, and foster professional growth, such as autonomy, performance feedback, equitable compensation, and career development opportunities. When employees face excessive job demands without adequate compensatory resources, they must expend additional energy to meet their work expectations. Prolonged resource depletion can erode motivation and organizational commitment, ultimately contributing to adverse psychological outcomes such as anxiety and depression. Fadare et al. demonstrated how pharmacists’ burnout stems from workload, work–family conflict, and insufficient job control and social support [25]. Klusmann et al. revealed how teachers’ well-being is mediated by work–life balance, administrative policies, and collegial relationships [26]. This study employs the JD-R model to systematically examine the aetiology of psychological distress among healthcare workers.

(3) Dissipative structure theory

This theory suggests that open systems that are far from equilibrium can generate self-organization phenomena through internal interactions by continuously exchanging matter and energy with the outside world. When a certain threshold is reached, the system can spontaneously transform from its original disordered state to a macroscopic ordered state in terms of time, space, and function, resulting in the formation of a new stable structure [27]. Although the theory of dissipative structures originated in the field of physical chemistry, its core idea—that open systems that are far from equilibrium can spontaneously form complex and ordered structures—provides a valuable perspective for understanding the psychological balance and adaptability of individuals in high-pressure environments [28]. The human psychological system is a complex whole composed of subsystems such as psychological processes, personality psychology, and psychological states that work together. It has dissipative structural characteristics such as openness, detachment from the equilibrium, nonlinearity, and fluctuations [29]. Healthcare workers need effective resources and support systems to develop self-organized adaptive mechanisms to maintain psychological balance and professional efficacy. If medical staff cannot adapt well to the current state of work, small fluctuations will accumulate and become enormous fluctuations. When the negative impact exceeds the individual’s tolerance threshold, it causes the psychology to change from order to low order and disorder, leading to the occurrence of disease. Some scholars have applied this theory to identify psychological crises among college students and construct an early warning indicator system for such crises by incorporating correlation entropy [30]. Other scholars argue that the dissipative structure of self-organization reflects the process of psychological regulation and have utilized this concept to examine the impact of illness uncertainty on patients’ psychological state [31]. This study employs dissipative structure theory to deepen the understanding of changes in individuals’ psychological processes.

### 2.2. Theoretical Integration and Application

The epidemiological triangle model attributes healthcare workers’ psychological health risks to environpsychological, host, and pathogen factors, offering a broad framework for identifying risks. However, its coarse granularity requires refinement for specific research questions. The job demands–resources (JD-R) model, a classic occupational stress theory, helps analyse healthcare work environments and characteristics, as well as how they impact psychological outcomes. From a systems perspective, dissipative structure theory aids in understanding the components, organization, and processes of the psychological system, thereby underscoring the role of individual self-regulation.

Based on theoretical analysis and supported by interviews and the literature, this study identifies seven dimensions in the formation process of psychological health risks among healthcare workers. The social environment and organizational management dimensions correspond to environpsychological factors in the epidemiological triangle model [32,33]. The social environment dimension encompasses broader societal conditions, including social structure, economic trends, and public attitudes, all of which negatively influence healthcare practices. The environment shapes psychological and behavioural responses. Currently, China’s public hospital reforms are at a critical stage, with policies on insurance, drug pricing, and medical services profoundly affecting hospitals and staff. However, fundapsychological rights, including health, rest, and dignity, remain insufficiently protected [34]. Unrealistic public expectations of healthcare workers also undermine their professional agency. The organizational management dimension includes structure, workflows, and workplace culture [35]. Effective management not only supports operational efficiency but also can reduce psychological strain by creating supportive conditions. While the hierarchical model common in public hospitals offers clarity and compliance, it can also foster inefficiency, poor communication, and a lack of humanistic care.

The dimensions of job demands, negative events, and job resources correspond to pathogenic factors in the epidemiological model [36,37]. Job demand risk entails excessive workload intensity, emotional labour demands, and performance evaluation pressures that frequently surpass an individual’s coping capacity [38]. Conversely, insufficient job resource risk reflects deficits in professional support systems and career advancement opportunities and is closely associated with job insecurity [39]. Job demands and job resources constitute the core elements of the JD-R model, representing established dimensions of occupational characteristics. Beyond these dimensions, this study introduces the negative event risk dimension. According to Goodyer, negative events refer to perceived unpleasant experiences whose accumulation may lead to illness [40]. Other scholars characterize them as significant occurrences within a given timeframe that exceed an individual’s capacity to manage or process [41]. In the present research, the negative event dimension encompasses various adverse situations that healthcare workers encounter in their roles, such as verbal aggression, occupational injuries, and complaints. These incidents often elicit pronounced feelings of frustration and helplessness. Unlike job demands and resources, negative events typically arise with greater suddenness [42]. Healthcare is fundamentally a helping profession, requiring practitioners to navigate diverse and often demanding situations. However, this professional responsibility can contribute to profound and sustained psychological strain.

Psychological adjustment corresponds to the host factor and reflects the self-organizing principle of dissipative structure theory [43]. It refers to challenges in regulating one’s psychological state under stress and involves how individuals recognize, process, and respond to pressures [44]. In a rapidly changing environment, impactful events can disrupt psychological equilibrium. Without effective adjustment, accumulated stress may lead to a shift from order to exhaustion, highlighting the role of individual agency in psychological well-being.

Finally, individual psychological health status serves as the outcome variable, capturing manifestations such as emotional disturbances, including burnout, anxiety, and depression, as well as negative behavioural intentions such as withdrawal and turnover. These manifestations reflect the overall impact of the identified risk dimensions on healthcare workers’ psychological health.

### 2.3. Research Hypotheses and Model

(1) Social environment risks, job demand risks, negative event risks, and job resource risks

With an ageing population, evolving family structures, and a growing chronic disease burden, public health needs and medical service standards are continuously changing, placing new demands on healthcare professionals’ roles and workflows [45]. Without sufficient government support for staffing, funding, and policy, healthcare delivery may suffer severe disruptions, a reality reflected in the fact that approximately 80% of developing countries report having inadequate financial resources [46]. Rapid systemic changes, such as shifts in payment models, the adoption of electronic health records, and the integration of new medical technologies, may further increase the daily workload of medical staff [47]. Moreover, rising public expectations for quality of life and stronger rights awareness often lead to an oversight of the inherent uncertainties and complexities in clinical practice. Healthcare workers, whose roles are distinct from those of general service professionals, are frequently judged by comparable standards, and unfavourable outcomes are attributed to them. Such one-sided perceptions fuel communication barriers and increase the risk of patient–provider conflict. Widespread public distrust towards the healthcare system also prompts more defensive medical practices, adding to the intensity and difficulty of clinical work. Public health crises, such as the COVID-19 pandemic, compound these challenges by creating sudden surges in demand and exposing healthcare workers to prolonged high-intensity workloads. Concurrent shortages of essential supplies such as masks and protective gear further exacerbate occupational risks and psychological stress. Accordingly, this study proposes the following hypotheses:

**H1.**  *Social environmental risks have a direct effect on job demand risks.*

**H2.**  *Social environmental risks have a direct effect on negative event risks.*

**H3.**  *Social environmental risks have a direct effect on job resource risks.*

(2) Social environment risks and organizational management risks

Various societal factors, such as economic, political, cultural, and legal conditions, influence the management and operation of healthcare institutions. Research by West et al. indicates that unfavourable public perception can heighten organizational risks for healthcare providers, such as challenges in human resource management and a decline in workplace conditions [48]. This direct influence of the external environment on internal operations aligns with socioecological systems theory. Healthcare institutions must continually adapt to evolving policies and regulations, while resource allocation also shapes their functioning, impacting staff compensation and career prospects. Studies using SAF regression further confirm that the societal environment significantly influences the operational efficiency of healthcare institutions in China, with insufficient government health spending reducing efficiency [49]. Accordingly, this study proposes the following hypothesis:

**H4:**  *Social environmental risks have a direct effect on organizational management risks.*

(3) Organizational management risks and job demand risks, negative event risks, and job resource risks

Research increasingly highlights the critical role of organizational factors in supporting healthcare professionals’ effective performance. Organizational management shapes job demands and allocates resources, directly influencing operational efficiency and quality of care. Inadequate systems or poor coordination of human, material, and financial resources can undermine work outcomes and increase challenges for staff. Studies have shown that ineffective management and poorly structured work arrangements contribute significantly to physician stress [50]. Leadership also affects individuals by shaping job demands and resources, as noted in a review of 139 publications [51]. Health-oriented leadership, in particular, can improve healthcare workers’ motivation and well-being [52]. Communication failures are linked to adverse events, with one analysis reporting a coefficient of determination of −0.32 [53]. Adequate equipment and resource support are essential for protecting frontline staff and maintaining service continuity, while poor financial planning can limit key initiatives and foster frustration over career development [54]. Accordingly, this study proposes the following hypotheses:

**H5.**  *Organizational management risks have a direct effect on job demand risks.*

**H6.**  *Organizational management risks have a direct effect on negative event risks.*

**H7.**  *Organizational management risks have a direct effect on job resource risks.*

(4) Job demand risks, negative event risks, job resource risks, and psychological health status

Existing theories and research confirm that job demands significantly affect psychological health. Healthcare professionals facing heavy workloads, frequent evaluations, high altruistic expectations, technical complexity, and work–family conflict are especially susceptible to psychological strain and energy depletion, which is consistent with the health impairment pathway of the JD-R model. Moreover, low compensation, limited career advancement, and similar constraints often hinder goal attainment. Repeated setbacks can trigger negative work attitudes through the motivational impairment process of the JD-R model. When compounded by adverse events, these persistent pressures progressively deplete psychological energy. Drawing on dissipative systems theory, a continuous influx of external stressors can result in the accumulation of psychological fluctuations, eventually leading to destabilization or psychological health disorders. Empirical studies support these dynamics: Olajide et al. linked heavy workload and work–family conflict to burnout among healthcare workers [25]. In a review of 20 studies, Jun et al. found that depersonalization among nurses correlated with increased falls and medication errors [55]. Perceived effort–reward imbalance is associated with anxiety symptoms [56], and insufficient job resources have been positively linked to suicidal ideation in this population [57]. Accordingly, this study proposes the following hypotheses:

**H8.**  *Job demand risks have a direct effect on psychological health status.*

**H9.**  *Negative event risks have a direct effect on psychological health status.*

**H10.**  *Job resource risks have a direct effect on psychological health status.*

(5) Psychological adjustment risks and psychological health status

The demanding nature of healthcare work places professionals in sustained high-pressure settings, increasing their psychological vulnerability. Within the field dynamics framework, ineffective personal adjustment functions as a negative force, generating stress and tension in one’s psychological environment. Some healthcare workers idealize their role, wrongly associating negative emotions with professional failure or fearing that seeking support could harm their reputation, which are attitudes that exacerbate burnout [58]. A study indicates that modifying nurses’ negative interpretations of work stressors can meaningfully alleviate stress reactions [59]. Multiple studies have also shown that healthcare personnel who rely predominantly on passive coping strategies during health crises are at increased risk for psychological difficulties [60]. Accordingly, this study proposes the following hypothesis:

**H11.**  *Psychological adjustment risks have a direct effect on psychological health status.*

(6) Psychological adjustment risks and job demand risks, negative event risks, job resource risks, and psychological health status

Drawing on dissipative systems theory, self-organizing individuals shape the evolution of open systems. Psychological adjustment serves as a mediator between internal states and external demands, reflecting one’s capacity for self-organization while influencing what enters and emerges from the psychological system. When increasing job demands, sudden adverse events, or persistent resource shortages introduce positive entropy into this system, early recognition and adaptive responses can reduce harm. Conversely, if an individual’s coping capacity is exceeded by the intensity or duration of stressors, psychological order may break down, resulting in psychological health impairment. Empirical research using the JD-R model indicates that personal resources partially mediate how job demands and resources affect health outcomes [61]. Such resources can also exhibit a spiral effect, partly explaining the link between job characteristics and burnout [62]. External resources further contribute to shaping internal resources. According to cumulative risk theory, exposure to multiple risk factors increases the likelihood of negative outcomes more than exposure to any single factor does [63]. Sustained high job demands and chronic stress not only reduce work performance but also may weaken immune function and diminish adaptive capacity. Extended work hours and frequent overtime can erode social connections, reducing perceived support and belonging. These factors compromise stress resilience and heighten vulnerability to psychological difficulties. Accordingly, this study proposes the following hypotheses:

**H12.**  *Psychological adjustment risks mediate the relationship between job demand risks and psychological health status.*

**H13.**  *Psychological adjustment risks mediate the relationship between negative event risks and psychological health status.*

**H14.**  *Psychological adjustment risks mediate the relationship between job resource risks and psychological health status.*

**Figure 1:**
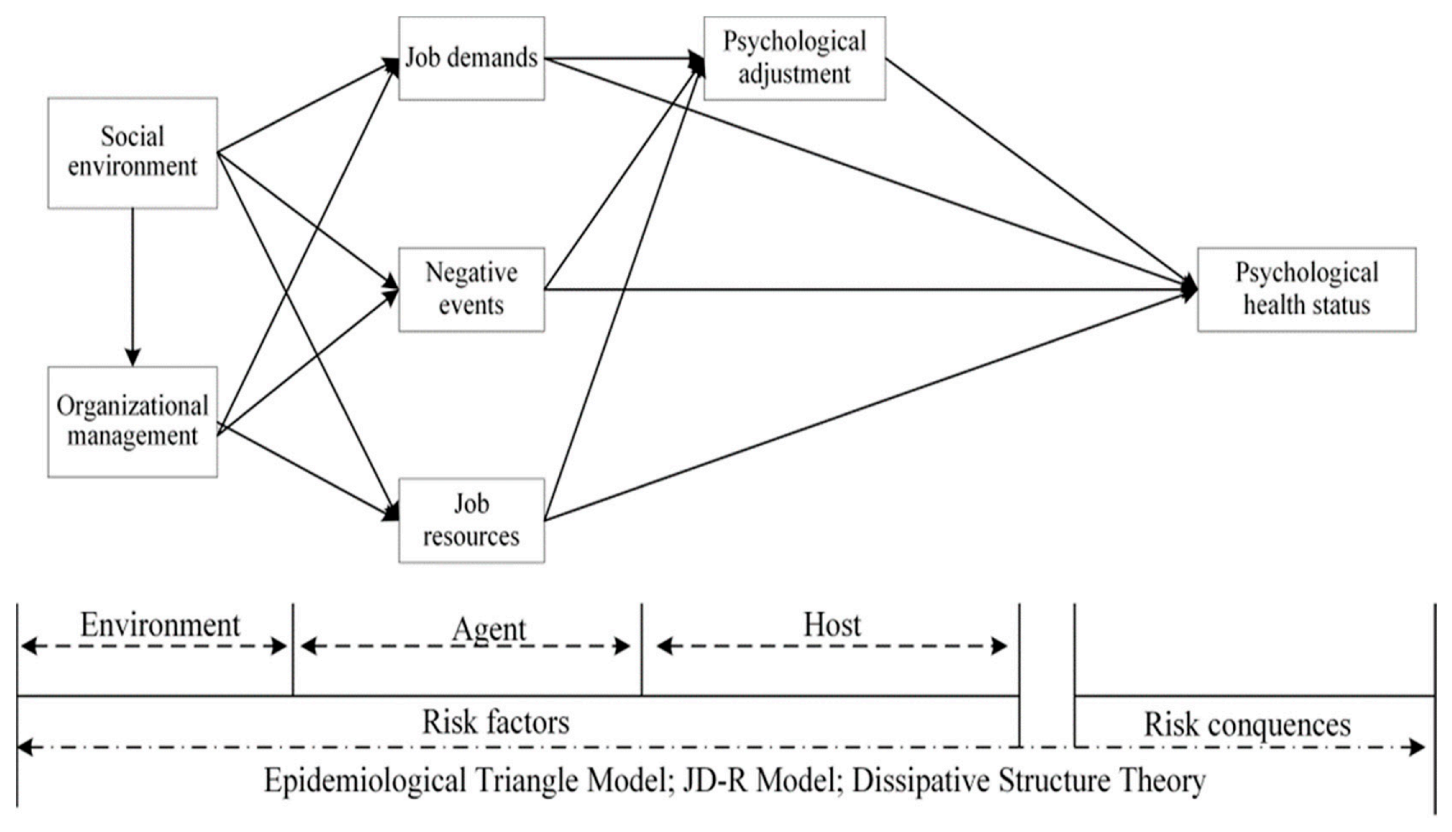
Research model.

Based on the above analysis, combined with the perspective of risk sources and transmission chains, this study assumed that social environpsychological risks and organizational management risks are remote risks that indirectly affect psychological health by determining the characteristics of medical work, with social environpsychological risks playing a fundapsychological role. Job demand risks, negative event risks, and job resource risks are proximal risks that affect an individual’s psychological health status directly and through the mediating role of psychological adjustment. The theoretical model of the formation mechanism of psychological health risks among medical staff is shown below in Figure 1.

## 3. Methods

### 3.1. Data Collection

This study utilized a cross-sectional design to evaluate the hypothesized model and strictly adhered to the STROBE guidelines for reporting observational research. Prior literature indicates that healthcare workers in tertiary hospitals experience a higher incidence of psychological issues compared to those in other hospital tiers [64]. To improve sample representativeness, we employed convenience sampling to select four tertiary hospitals across several provinces in central China as survey sites, including two general hospitals, one specialized hospital, and one traditional Chinese medicine hospital, capturing variations in service orientation and organizational culture among healthcare settings. Each participating hospital contributed a minimum of 200 responses. Our study focused on clinical departments, such as internal medicine, surgery, obstetrics and gynecology, emergency, and critical care. Questionnaires were administered using a blended online and offline approach to accommodate diverse work routines and accessibility among healthcare workers, thereby mitigating potential coverage bias associated with a single survey method. Eligible participants included practicing medical staff who provided informed consent, while interns, trainees, and those unwilling to participate were excluded. To ensure data quality, all questionnaires emphasized anonymous participation without performance consequences to encourage truthful responses. For offline administration, researchers received standardized training to properly administer the surveys and to provide accurate guidance to the participants. Completed offline questionnaires were checked on-site for any omissions, and participants were asked to provide responses promptly to ensure completeness before submission. Online surveys were distributed via Wenjuanxing QR codes with support from hospital leadership, including vice deans of research, department heads, and nursing supervisors, who encouraged the participation of clinical staff on the basis of actual work experience. The online platform incorporated conditional restrictions to ensure complete responses, while offline questionnaires underwent rigorous double-entry verification via EpiData 3.1 to maintain data accuracy throughout the collection process.

A total of 1014 questionnaires were collected. Questionnaires that were completed too quickly or had completely consistent answers were excluded. Finally, a total of 930 valid questionnaires were obtained, for a valid response rate of 91.72%.

### 3.2. Data Analysis

In this study, the associations between psychological health risk factors and related outcomes among healthcare workers were examined via structural equation modelling (SEM), a method well-suited for analysing complex intercorrelational structures involving multiple latent variables. The analysis utilized data from 930 valid questionnaires collected in the formal survey, exceeding the recommended sample size requirement of 10–15 observations per latent variable. We conducted SEM analysis in AMOS 26.0, employing the following fit criteria: χ^2^/df < 3 (or acceptable up to 5), RMSEA < 0.08, and GFI, AGFI, NFI, CFI, and IFI > 0.90 [65]. To test the path effects, we performed bias-corrected nonparametric bootstrap analysis with 5000 resamples, considering effects to be statistically significant when the 95% confidence intervals excluded zero.

To assess model robustness, we conducted key variable substitution by replacing general psychological health measures with specific risk outcomes: burnout, anxiety, depression, suicidal ideation, and turnover intentions. These five indicators reflect the psychological health status of healthcare workers and also represent common and urgent concerns that medical institutions must address. Burnout was measured via the 5-item Emotional Exhaustion subscale of the MBI-GS (score range: 0–30; α = 0.965), which captures work-related stress and fatigue as the core dimension of burnout [66]. Anxiety symptoms were assessed with the GAD-7 (7 items; 0–21 points; α = 0.961), which evaluates the frequency of anxiety-related experiences over two weeks [67]. Depressive symptoms were measured via the shortened 9-item CES-D (0–27 points; α = 0.925), which assesses depressive experiences during the previous week [68]. Suicidal ideation was evaluated through a single-item measure based on the criteria of Liu et al., scored on a 5-point scale reflecting thought frequency over the past year [69]. Turnover intentions were measured via Zhang’s 3-item scale (1–5 points per item), with higher scores indicating a greater likelihood of resignation [70].

In addition to variable substitution, we applied the multiple indicators, multiple causes (MIMIC) approach to examine the robustness of the hypothesized model. After accounting for these variables, the MIMIC model allowed us to assess the influence of key covariates on the structural relationships while simultaneously testing the stability of the path coefficients after accounting for these variables. Based on the literature and preliminary findings, we included the participants’ age, gender, occupation type, department affiliation, education level, professional rank, marital status, years of work experience, income level, and self-rated physical health as covariates in the analysis.

## 4. Results

The final sample comprised 930 valid responses, including 430 males (46.2%) and 500 females (53.8%). The majority of participants were under 40 years old and held bachelor’s degrees. With respect to professional titles, junior and intermediate levels were most common. The sample showed a balanced representation between physicians (51.4%) and nurses (48.6%). Most respondents reported a monthly income of 5000–10,000 CNY and rated their physical health as average. The detailed demographic characteristics are presented in Supplementary Material Table S1.

### 4.1. Structural Equation Modelling Results

The questionnaire results revealed varying risk levels across different dimensions. Social environment risks (M = 3.95, SD = 0.88) and job demand risks (M = 3.71, SD = 0.96) had the highest scores, followed by organizational management risks (M = 3.42, SD = 1.11) and job resource risks (M = 3.27, SD = 1.06). Negative event risks had a relatively lower score (M = 3.06, SD = 1.09), as did psychological adjustment risks (M = 3.11, SD = 0.79). The participants’ average psychological health status was M = 3.18, SD = 1.04, indicating moderate levels of psychological well-being.

**Figure 2:**
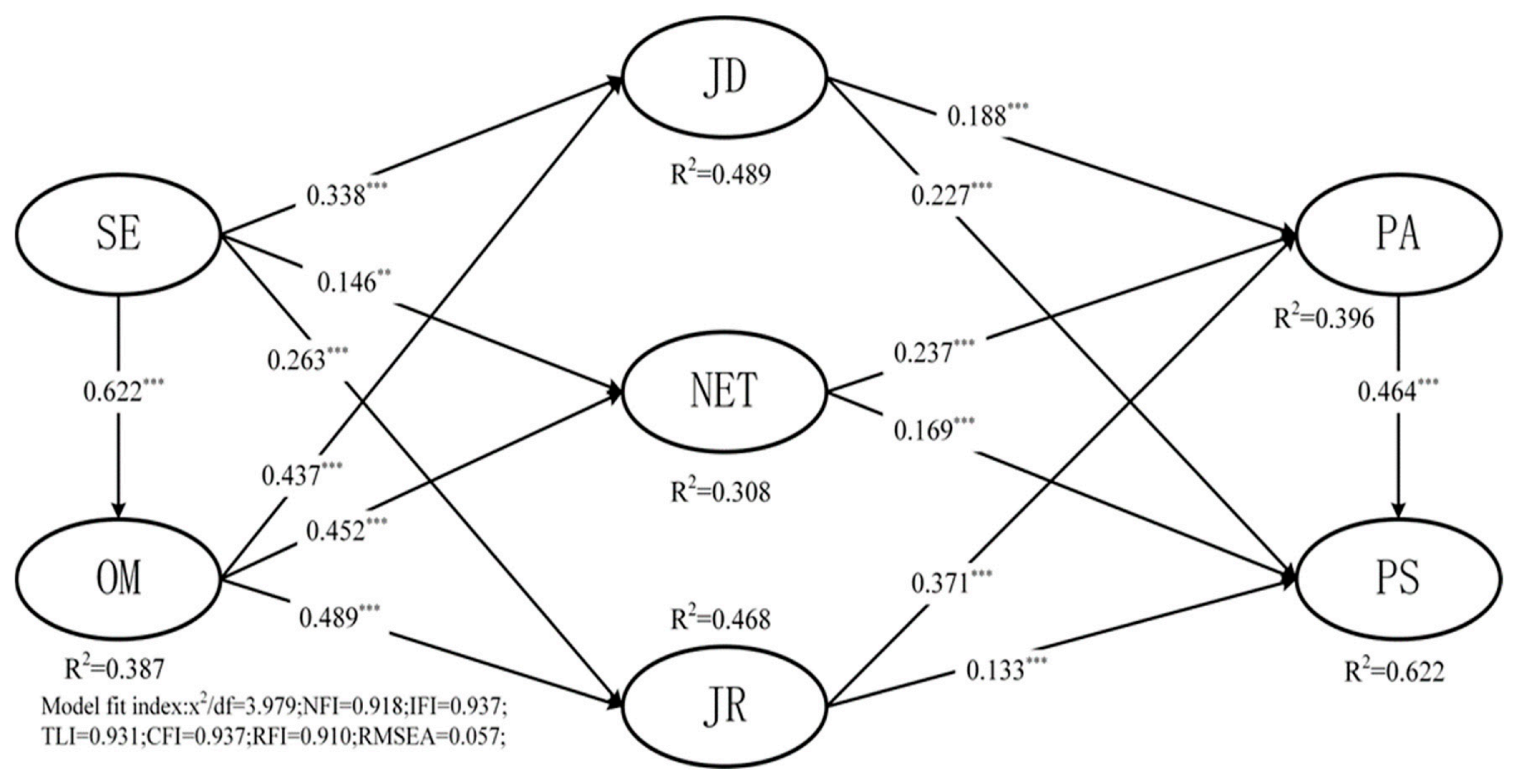
SEM results. Note: The standardized path coefficients are presented. * *p* < 0.05; ** *p* < 0.01; *** *p* < 0.001. SE: social environment risks; OM: organizational management risks; JD: job demand risks; NET: negative event risks; JR: job resource risks; PA: psychological adjustment risks; PS: psychological health status.

Figure 2 presents the structural equation modelling results, which demonstrate an acceptable model fit ($\chi^{2}/df$ = 3.979; NFI = 0.918; IFI = 0.937; TLI = 0.931; CFI = 0.937; RFI = 0.910; RMSEA = 0.057), with all hypothesized paths reaching statistical significance (*p* < 0.05). The model explained substantial variance in the endogenous variables (R^2^ = 0.622) and revealed complex mediation patterns. As initially assumed, social environmental risks (*β* = 0.622) directly affected organizational management risks (R^2^ = 0.387). Furthermore, social environmental risks (*β* = 0.338) and organizational management risks (*β* = 0.437) directly affected job demand risks (R^2^ = 0.489). Social environmental risks (*β* = 0.146) and organizational management risks (*β* = 0.452) directly affected negative event risks (R^2^ = 0.308). Social environmental risks (*β* = 0.263) and organizational management risks (*β* = 0.489) directly affected job resource risks (R^2^ = 0.468). On the one hand, job demand risks (*β* = 0.227), negative event risks (*β* = 0.169), and job resource risks (*β* = 0.133) directly affected individuals’ psychological health. On the other hand, job demand risks (*β* = 0.188), negative event risks (*β* = 0.237), and job resource risks (*β* = 0.371) indirectly affected psychological health by influencing psychological adjustment (R^2^ = 0.396). More detailed results are shown in Supplementary Material Table S2.

The effect size analysis revealed distinct patterns across risk factors. The nonstandardized effect sizes were as follows: social environment risks (0.501), organizational management risks (0.384), job demand risks (0.313), negative event risks (0.236), job resource risks (0.271), and psychological adjustment risks (0.516). The corresponding standardized effects were 0.484, 0.412, 0.314, 0.279, 0.305, and 0.464, respectively. With respect to direct effects, psychological adjustment risks showed the strongest effect (0.464), followed by job demands (0.227), negative events (0.169), and job resources (0.133). Indirect effects were most substantial for the social environment (0.484) and organizational management (0.412), with job demands (0.087), negative events (0.110), and job resources (0.172) demonstrating smaller but significant mediating effects. The complete effect size details are presented in Supplementary Material Table S3.

### 4.2. Structural Equation Modelling Results with Key Variable Replacement

**Figure 3:**
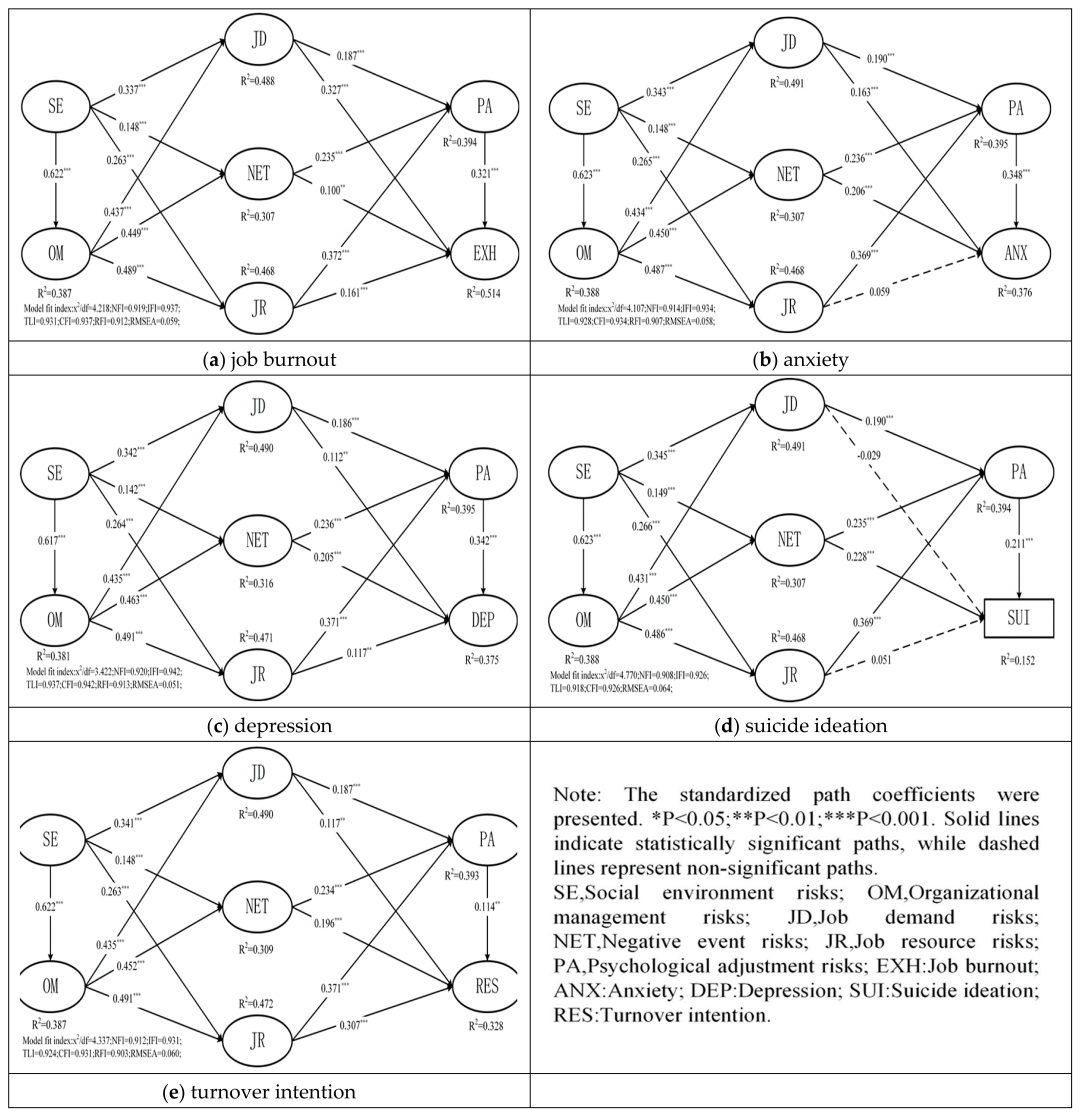
SEM results with key variable replacement.

The study employed validated scales to assess psychological outcomes, revealing moderate levels of job burnout (14.51 ± 7.946), anxiety (6.72 ± 5.901) and depression (8.95 ± 6.648). The participants reported mean scores of 1.68 ± 1.037 for suicidal ideation and 8.47 ± 3.606 for turnover intention. As depicted in Figure 3, the alternative outcome model analysis revealed that while most pathways remained statistically significant, two exceptions emerged: job demands showed no significant direct effect on anxiety, and neither job demands nor job resources demonstrated significant direct effects on suicidal ideation. The model exhibited considerable explanatory power, accounting for 51.4%, 37.6%, 37.5%, 15.2%, and 32.8% of the variance in burnout, anxiety, depression, suicidal ideation, and turnover intentions, respectively, with all effect sizes falling within medium to large ranges. These results substantiate the theoretical importance of the six risk dimensions and provide empirical support for the robustness of the proposed mental health risk model among healthcare workers.

The analysis revealed distinct patterns in standardized effect sizes across psychological outcomes. With respect to job burnout, social environment risks (0.470), job demand risks (0.387), and organizational management risks (0.385) demonstrated particularly strong effects, whereas negative event risks had a more modest influence (0.175). Direct effects consistently exceeded indirect effects for job demands (0.327 vs. 0.060), negative events (0.100 vs. 0.075), and job resources (0.161 vs. 0.119). Anxiety outcomes were most strongly predicted by social environmental risks (0.370) and psychological adjustment risks (0.348). For depression, effect sizes ranged from 0.175 (job demands) to 0.367 (social environment), with job resources having stronger indirect effects (0.127) than direct effects (0.117). Negative event risks emerged as the strongest predictor of suicidal ideation (0.278), followed by psychological adjustment risks (0.211). Turnover intentions were most influenced by social environmental risks (0.379) and job resource risks (0.350), with psychological adjustment risks having a minimal effect (0.114). Complete statistical details are provided in Supplementary Material Table S4.

In summary, the analysis revealed that social environmental risks exerted the strongest influence across all adverse psychological health outcomes except suicidal ideation. Psychological adjustment emerged as the second most significant factor in psychological health risk formation, following social environmental risks. Notably, negative event risks played a determining role in suicidal ideation. Job demands and negative event risks demonstrated substantially stronger direct effects than indirect effects. Job resources showed comparable direct and indirect effects for all outcomes except turnover intentions. Table 1 presents the standardized effect sizes of all risk factors with their relative rankings following key variable substitution.

### 4.3. Multiple Indicators, Multiple Causes (MIMIC) Results

After the covariates were added, the fit of the MIMIC model was acceptable: $\chi^{2}/df$ =3.556, TLI = 0.902, CFI = 0.914, and RMSEA = 0.052. The structural relationships among the variables in the psychological health risk model remained largely consistent, with only minor variations in the strength of the estimated associations.

## 5. Discussion

Healthcare professionals serve as primary providers of medical services and constitute a crucial pillar of the healthcare system. Enhancing their psychological well-being has significant implications for improving service quality, increasing patient satisfaction, and optimizing resource allocation within healthcare systems. While traditional health risk research focuses primarily on identifying the factors that contribute to adverse physical outcomes, this study addresses the critical issue of mental health risks among medical staff. By adopting a risk management perspective, we developed an integrated theoretical framework encompassing seven key constructs to explain the formation of psychological health risks in this population. The fitted model further revealed the intercorrelational structure among the risk factors and provided estimates of the strength of associations between each risk factor and the psychological health outcomes.

The study revealed significantly elevated risk levels in terms of both the social environment (3.95 ± 0.88) and job demands (3.71 ± 0.96), corroborating previous findings by Li Huaiyong et al., who highlighted frequent performance evaluations (2.49 points) and excessive societal expectations (2.03 points) as predominant stressors for medical professionals [71]. Comparative analysis of environmental factors revealed that external social pressure substantially exceeded internal organizational risks (3.42 ± 1.11), underscoring the disproportionate psychological burden imposed by societal factors on healthcare workers. This finding aligns with the established literature documenting how public moral expectations and professional role constraints contribute to clinician burnout [72,73]. Notably, while organizational management risks appeared to be lower overall, the considerable standard deviation (1.11) reflects substantial variation in workplace experiences, indicating unmet organizational support needs among certain medical staff subgroups.

Drawing on academic reports from Safe Work Australia, this study categorized occupational risks into three dimensions: job demands, job resources, and negative events [74]. The empirical results revealed that job demands were the greatest risk factor (3.71 ± 0.96), followed by job resources (3.27 ± 1.06) and negative events (3.06 ± 1.09). High job demand scores reflect significant psychological health stressors, including excessive workloads and prolonged working hours among healthcare professionals. Emerging evidence suggests that patient care complexity may represent an important upstream determinant of healthcare workers’ occupational burden [75]. Although negative events are less frequent, which accounts for their lower scores, they still retain substantial potential for severe and lasting psychological impact [55,76]. Medical staff demonstrated moderate psychological adjustment capacity (3.11 ± 0.79), suggesting generally effective but improvable coping strategies [77]. These findings align with the literature indicating that healthcare professionals typically possess better mental health literacy than the general population does, with problem-solving representing their predominant coping mechanism. Moreover, consistent with previous findings, medical staff predominantly employ problem-solving coping strategies, where individual factors play a crucial moderating role in mitigating mental health risks [78].

The SEM results demonstrated that both the social environment risk dimension and the psychological adjustment risk dimension were significantly associated with psychological health. The social environment had an unstandardized effect value of 0.501 and a standardized effect value of 0.484, whereas psychological adjustment had an unstandardized effect of 0.516 and a standardized effect of 0.464. Notably, these effect sizes were closely comparable, highlighting the considerable role of social environmental factors alongside individual psychological adjustment in shaping psychological health risks among medical staff. This point was further supported by the relatively high survey score for social environmental risks (3.95, with a standard deviation of 0.88). Within the Chinese cultural context, which emphasizes collectivism and social evaluation, medical staff may be particularly sensitive to distrust from patient populations and moral pressure. Meanwhile, the current reform of public hospitals is entering a critical phase, and the tiered diagnosis and treatment system remains imperfect, rendering both doctors and patients susceptible to role misalignment under high workloads [5,71]. During the interviews, medical staff frequently emphasized the growing severity of doctor–patient conflicts compared with the planned economy era, as well as increasing societal expectations that sometimes manifested as moral pressure or excessive demands. Such proactive societal influences, including widespread distrust and adversarial perceptions, underscore the urgent need for systemic improvements within the medical ecosystem. Furthermore, our analysis supports the buffering mode hypothesis, which posits that psychological adjustment mitigates the impact of pathogenic factors such as job demands, negative events, and job resources on psychological health. This result aligns with previous studies by Zhou [79], Hall [80], and Zhang [81]. Importantly, these findings suggest practical interventions for healthcare administrators. By implementing targeted training programmes to enhance psychological adjustment skills, institutions may reduce the prevalence of psychological distress among medical staff.

The study demonstrated significant effect values for three pathogenic factors influencing psychological health: job demands (0.314), negative events (0.279), and job resource risks (0.305). These findings strongly parallel those of Anders et al., who examined similar factors among police officers and reported effect values of 0.32 for organizational requirements, 0.31 for perceived resources, 0.14 for trauma exposure, and 0.30 for initiative [82]. While the study populations differed, both investigations focused on high-stress occupational environments and revealed consistent patterns regarding how job demands, resource availability, and critical incidents affect psychological health outcomes. The analysis indicated that job demands and negative events were associated with stronger direct effects than indirect effects, whereas job resources exhibited the opposite pattern. This distinction likely occurs because job demands and adverse events tend to be unpredictable and urgent, frequently placing healthcare workers in situations where they must react passively, thereby creating immediate psychological health consequences. In contrast, insufficient job resources primarily diminish medical professionals’ ability to handle occupational challenges, leading to psychological strain through the progressive accumulation of fatigue [83]. This mechanism explains why job resource limitations demonstrate more substantial indirect than direct effects on psychological health outcomes.

This study’s variable substitution analysis revealed significant effects across most pathways, except the effect of job resource risk on anxiety and the effect of both job demand risk and job resource risk on suicidal ideation. These nonsignificant relationships may reflect the fundamental psychological characteristics of anxiety and suicidal ideation while highlighting the crucial role of individual psychological adjustment. Anxiety stems from excessive worry about potential threats, particularly when people face unpredictable and uncontrollable job demands and negative events. Our finding of the direct effect of job demand risks on anxiety aligns with existing evidence: Italian researchers reported significantly higher anxiety scores among medical staff facing high job demands (2.33 vs. 1.94, *p* < 0.05) [84], and data from the Philippines similarly revealed that healthcare workers with high job demands had 2.71 times greater odds of anxiety symptoms (95% CI: 1.71–4.29) [85]. These consistent findings across cultures demonstrate how intensive job demands, time pressure, and task complexity collectively increase psychological burdens and precipitate anxiety, particularly when individuals’ personal coping capacity is exceeded.

With respect to suicidal ideation, despair represents its core psychological feature. Negative events were associated with intense emotional responses and overwhelmed coping mechanisms, which were linked to a higher likelihood of extreme solutions [86]. Humans possess innate adaptive psychology, yet chronic exposure to excessive job demands and resource scarcity may induce learned helplessness [87]. When this vulnerable psychological state coincides with acute negative events, cumulative stress may reach a critical threshold at which adverse incidents become the precipitating factor for suicidal behaviour.

The analysis revealed distinct effect sizes for different risk dimensions influencing turnover intentions among medical staff. Job resource risks had a substantial effect (0.350), whereas psychological adjustment risks had a comparatively modest effect (0.114). These findings indicate that medical professionals prioritize work resource considerations over psychological factors when they make decisions about resignation. This observation supports the principles of conservation of resources theory, which proposes that individuals possess finite resources and actively seek to protect those that they value most. Numerous previous studies have confirmed that employees who perceive inadequate treatment within an organization frequently choose to leave [88]. The relatively low effect of psychological adjustment risks further indicates that resigning employees predominantly attribute their decisions to external organizational factors rather than internal psychological factors. This aligns with the findings of AL-Abrrow et al., whose study demonstrated that the severe depletion of job resources during the COVID-19 pandemic led to an increase in turnover intention among healthcare workers [89].

The analysis revealed differential effect sizes across risk dimensions, with all six dimensions collectively explaining moderate to substantial variance in outcomes, as demonstrated by the fact that R squared values ranged from 0.152 to 0.622. These results clearly indicate that the identified risk factors significantly contribute to the emergence of psychological health problems. The MIMIC model demonstrated good stability after covariate adjustment. The structural relationships among the dimensions remained largely consistent, with only minimal changes in the magnitude of the estimated coefficients. These findings support the potential development of differentiated intervention strategies tailored to specific risk factor profiles, offering significant practical applications for implementing targeted mental health prevention approaches.

## 6. Implications for Theory and Practice

To the best of our knowledge, this study represents one of the first systematic investigations of psychological health risk factor interactions among healthcare workers. In existing research, comprehensive analyses of psychological health risk factors specific to medical staff are lacking. The occurrence of psychological issues among healthcare professionals is not attributable to a single cause but rather results from the interplay of multiple factors [90]. To address this gap, we developed an integrated theoretical framework by combining the epidemiological triangle model, the job demands–resources model, and dissipative structure theory. This framework offers a conceptual lens for exploring the interrelated risk factors associated with psychological health in healthcare workers. The empirical testing of this framework extends current theoretical perspectives in hospital management and occupational health psychology.

From a theoretical development perspective, this study extends the epidemiological triangle and dissipative structure theories by enhancing their explanatory power and broadening their applicability. Although the JD-R model is well established in occupational stress research, its scope has largely been confined to the direct effects of job characteristics on psychological health. Our study expands this model by incorporating psychological adjustment as a key individual resource; positioning organizational management and the social environment as contextual antecedents to job characteristics; and further refining the classification of job characteristics into distinct categories, namely, job demands, adverse events, and job resources, based on their inherent risk attributes.

From a practical standpoint, psychological health risk management for healthcare workers should adopt a systematic, multitiered intervention approach based on the empirical findings. The social environment, as the most influential distal factor, necessitates collective societal engagement. The media should adopt balanced and responsible reporting to avoid sensationalism that exacerbates tensions between doctors and patients, while public education can foster realistic professional expectations, reduce moral stigmatization, and cultivate a climate of respect for healthcare workers [91]. Simultaneously, policy development should focus on strengthening legal protections for medical staff, lowering barriers to safeguarding their rights, and increasing penalties for violations in healthcare settings, thereby establishing a solid legal foundation for occupational safety.

At the organizational level, hospital management should introduce scientific workload assessment and dynamic staffing models, particularly in high-pressure departments such as emergency and intensive care, coupled with rigorous oversight of overtime. The results further highlight the potential importance of job resources, including career development opportunities, compensation equity, and clinical autonomy, in relation to the psychological correlates of effort-reward imbalance [92]. Organizations should also proactively address interpersonal conflicts and establish structured support mechanisms, including confidential counselling services, digital psychological health platforms, and peer support programs, to better manage adverse events without overreliance on individual coping [93].

Given the central mediating role of psychological adaptation, regular training in stress management, emotional regulation, and communication should be incorporated into professional development to strengthen psychological resilience. Improving psychological health literacy further enables staff to recognize occupational risks and effectively utilize available support, thereby buffering the impact of work-related stressors [94]. Beyond institutional measures, cross-sector collaboration is essential for establishing a nationwide psychological health monitoring and early warning system for healthcare workers. Such collaboration should be led by health authorities and actively involve medical, academic, and research institutions. Increased investment in occupational health is also needed to advance the design and implementation of evidence-based interventions. Sustained improvement in healthcare workers’ professional well-being can be achieved only through an integrated approach that combines refined internal organizational management with robust external institutional safeguards and societal support [95].

## 7. Limitations

This study has several limitations that should be acknowledged. First, the cross-sectional design prevented the establishment of definitive causal relationships between risk factors and psychological health outcomes, necessitating future longitudinal studies to clarify these temporal associations. Second, this study focused on staff from tertiary hospitals in China. While this focus allowed an in-depth examination of a population with known psychological vulnerability, it limits the generalizability of our findings to healthcare workers in other settings, such as primary or secondary care institutions, and to other cultural contexts. Future research across diverse medical institutions and cultures is needed to validate and compare these findings. Third, although we implemented methodological safeguards, including reverse-scored items, and guaranteed anonymity, the self-reported nature of the data remains susceptible to recall and reporting biases. Future studies should incorporate multisource data to enhance objectivity.

## 8. Conclusions

This study developed a comprehensive theoretical framework for psychological health risks among medical staff. An analysis of 930 validated questionnaires revealed significantly higher risk scores for the social environment and job demands than for negative events and psychological adjustment. The empirical findings largely supported our hypothesized framework. Most of the proposed relationships were statistically significant, except for the link between job resources and anxiety and the links of job demands and job resources with suicidal ideation. These findings underscore that effective psychological health promotion must evolve from isolated support programs into an integrated, multi-level synergistic system.

## Figures and Tables

**Table 1:** Standardized SEM coefficients for risk factor—outcome associations.

Risk Factors	Risk Consequences (Individual Psychological Health Status)					
	Psychological Health (Self-Developed)	Job Burnout (Validated Scale)	Anxiety (Validated Scale)	Depression (Validated Scale)	Suicide Ideation (Validated Scale)	Turnover Intention (Validated Scale)
**Social environment**	① 0.484	① 0.470	① 0.370	① 0.367	③ 0.199	① 0.379
**Organizational management**	③ 0.412	③ 0.385	③ 0.320	③ 0.328	④ 0.192	③ 0.332
**Job demands**	④ 0.314	② 0.387	⑤ 0.229	⑥ 0.175	0.011	⑤ 0.138
Direct effects	0.227 (72.29%)	0.327 (84.50%)	0.163 (71.18%)	0.112 (63.43%)	−0.029	0.117 (84.78%)
Indirect effects	0.087 (27.71%)	0.060 (15.50%)	0.066 (28.82%)	0.064 (36.57%)	0.040	0.021 (15.22%)
**Negative events**	⑥ 0.279	⑥ 0.175	④ 0.288	④ 0.286	① 0.278	④ 0.222
Direct effects	0.169 (60.57%)	0.100 (57.14%)	0.206 (71.53%)	0.205 (71.68%)	0.228 (82.01%)	0.196 (88.29%)
Indirect effects	0.110 (39.43%)	0.075 (42.86%)	0.082 (28.47%)	0.081 (28.32%)	0.050 (17.99%)	0.027 (12.16%)
**Job resources**	⑤ 0.305	⑤ 0.281	0.188	⑤ 0.244	0.129	② 0.350
Direct effects	0.133 (43.61%)	0.161 (54.65%)	0.059	0.117 (47.95%)	0.051	0.307 (87.71%)
Indirect effects	0.172 (56.39%)	0.119 (45.35%)	0.128	0.127 (52.05%)	0.078	0.042 (12.29%)
**Psychological adjustment**	② 0.464	④ 0.321	② 0.348	② 0.342	② 0.211	⑥ 0.114

Note: Circled superscript numerals denote the ranking of risk effect magnitudes.

## Data Availability

Data supporting the findings of this study are available from the corresponding author upon reasonable request. The data are not publicly available due to privacy and ethical restrictions.

## Supplementary Materials

The following supporting information can be downloaded at: https://www.mdpi.com/article/10.3390/healthcare14152280/s1, Table S1. Demographic characteristics. Table S2. SEM results. Table S3. Standardized SEM coefficients. Table S4. Standardized SEM coefficients (key variable replacement).

## Abbreviations

WHO	World Health Organization
COVID-19	Coronavirus Disease 2019
SEM	Structural Equation Modeling
JD-R	Job Demands–Resources Model
MIMIC	Multiple Indicators, Multiple Causes
STROBE	Strengthening the Reporting of Observational Studies in Epidemiology
