# Supplementary material for: Psychological Health Risk Factors Among Healthcare Workers: A Structural Equation Modeling Approach
Source: Healthcare (Basel). 2026 Jul 27;14(15):2280. doi: 10.3390/healthcare14152280 (PMC13466093; doi:10.3390/healthcare14152280)
Supplement: Supplementary file 1 [file healthcare-14-02280-s001.zip › healthcare-4380076-supplementary.pdf]

Supplementary Material

Table S1 Demographic characteristics

Characteristics	Categories	Number(N)	Percentage(%)
Gender	Male	430	46.24
	Female	500	53.76
Age (years)	20-30	342	36.77
	31-40	315	33.87
	41-50	168	18.06
	≥51	105	11.29
Occupation	Doctor	478	51.40
	Nurse	452	48.60
Department	Type A	399	42.90
	Type B	531	57.10
Education level	Doctoral degree	13	1.40
	Master’s degree	187	20.11
	Bachelor’s degree	634	68.17
	Junior college or below	96	10.32
Professional title	To be evaluated	57	6.13
	Junior	374	40.22
	Intermediate	317	34.09
	Associate senior	144	15.48
	Senior	38	4.09
Marital status	Unmarried	240	25.81
	Married	690	74.19
Years of work experience	≤5	242	26.02
	6-10	285	30.65
	11-20	202	21.72
	21-30	133	14.30
	＞30	68	7.31
Average monthly income (CNY)	＜5000	300	32.26
	5000-10000	524	56.34
	10001-15000	76	8.17
	>15000	30	3.23
Self-rated health status	Poor	242	26.02
	Average	443	47.63
	Good	245	26.34

Note: Type A departments refer to the Infectious Diseases Department, Emergency Department, Obstetrics and Gynecology Department, Pediatrics Department, Cardiology Department, Anesthesiology Department, Oncology Department, ICU (Intensive Care Unit), CCU (Coronary Care Unit), and Psychiatry Department; Type B departments refer to other departments.

Table S2 SEM results

Paths	Coefficients *β*	Standardised coefficients *S.β*	Standard error	*t* statistics	*P* value
SE→OM	0.693	0.622	0.040	17.221	＜0.001
SE→JD	0.352	0.338	0.041	8.621	＜0.001
SE→NET	0.179	0.146	0.053	3.278	0.001
SE→JR	0.307	0.263	0.045	6.778	＜0.001
OM→JD	0.409	0.437	0.037	11.127	＜0.001
OM→NET	0.496	0.452	0.049	10.194	＜0.001
OM→JR	0.512	0.489	0.042	12.311	＜0.001
JD→PA	0.168	0.188	0.033	5.164	＜0.001
JD→PS	0.226	0.227	0.031	7.209	＜0.001
NET→PA	0.180	0.237	0.027	6.694	＜0.001
NET→PS	0.143	0.169	0.026	5.566	＜0.001
JR→PA	0.296	0.371	0.031	9.679	＜0.001
JR→PS	0.118	0.133	0.029	4.055	＜0.001
PA→PS	0.516	0.464	0.044	11.790	＜0.001

Note: SE,Social environment risks; OM,Organizational management risks; JD,Job demand risks; NET,Negative event risks; JR,Job resource risks; PA,Psychological adjustment risks; PS,Psychological health status.

Table S3 Standardized SEM coefficients

Constructs	Total effects	95%CI Lower	95%CI upper	*P* value	Direct effects	95%CI Lower	95%CI upper	P value	Indirect effects	95%CI Lower	95%CI upper	*P* value
SE	0.484	0.419	0.551	0.003	—	—	—	—	0.484	0.419	0.551	0.003
OM	0.412	0.308	0.501	0.005	—	—	—	—	0.412	0.308	0.501	0.005
JD	0.314	0.221	0.400	0.005	0.227	0.148	0.306	0.004	0.087	0.033	0.239	0.004
NET	0.279	0.192	0.377	0.004	0.169	0.088	0.244	0.004	0.110	0.061	0.162	0.004
JR	0.305	0.196	0.400	0.004	0.133	0.032	0.223	0.021	0.172	0.117	0.147	0.002
PA	0.464	0.367	0.546	0.005	0.464	0.367	0.546	0.005	—	—	—	—

Note: SE,Social environment risks; OM,Organizational management risks; JD,Job demand risks; NET,Negative event risks; JR,Job resource risks; PA,Psychological adjustment risks; PS,Psychological health status.

Table S4 Standardized SEM coefficients (key variable replacement)

Risk consequences	Constructs	Total effects	95%CI Lower	95%CI upper	*P* value	Direct effects	95%CI Lower	95%CI upper	P value	Indirect effects	95%CI Lower	95%CI upper	*P* value
Job burnout	SE	0.470	0.416	0.530	0.003	—	—	—	—	0.470	0.416	0.530	0.003
	OM	0.385	0.290	0.467	0.004	—	—	—	—	0.385	0.290	0.467	0.004
	JD	0.387	0.300	0.466	0.006	0.327	0.247	0.298	0.004	0.060	0.023	0.105	0.003
	NET	0.175	0.093	0.249	0.004	0.100	0.026	0.328	0.011	0.075	0.042	0.114	0.004
	JR	0.281	0.180	0.371	0.004	0.161	0.048	0.248	0.004	0.119	0.075	0.180	0.002
	PA	0.321	0.230	0.398	0.006	0.321	0.230	0.398	0.006	—	—	—	—
Anxiety	SE	0.370	0.319	0.419	0.005	—	—	—	—	0.370	0.319	0.419	0.005
	OM	0.320	0.245	0.393	0.004	—	—	—	—	0.320	0.245	0.393	0.004
	JD	0.229	0.138	0.308	0.004	0.163	0.081	0.246	0.003	0.066	0.025	0.112	0.004
	NET	0.288	0.198	0.377	0.004	0.206	0.118	0.287	0.005	0.082	0.049	0.125	0.003
	JR	0.188	0.073	0.292	0.005	0.059	-0.071	0.164	0.375	0.128	0.078	0.193	0.003
	PA	0.348	0.249	0.421	0.008	0.348	0.249	0.421	0.008	—	—	—	—
Depression	SE	0.367	0.317	0.416	0.004	—	—	—	—	0.367	0.317	0.416	0.004
	OM	0.328	0.251	0.406	0.004	—	—	—	—	0.328	0.251	0.406	0.004
	JD	0.175	0.086	0.255	0.005	0.112	0.028	0.194	0.023	0.064	0.026	0.111	0.003
	NET	0.286	0.192	0.373	0.004	0.205	0.108	0.279	0.005	0.081	0.050	0.124	0.002
	JR	0.244	0.126	0.346	0.004	0.117	0.004	0.223	0.034	0.127	0.076	0.186	0.003
	PA	0.342	0.253	0.428	0.004	0.342	0.253	0.428	0.004	—	—	—	—
Suicide ideation	SE	0.199	0.163	0.243	0.002	—	—	—	—	0.199	0.163	0.243	0.002
	OM	0.192	0.137	0.243	0.005	—	—	—	—	0.192	0.137	0.243	0.005
	JD	0.011	-0.077	0.097	0.728	-0.029	-0.112	0.060	0.537	0.040	0.017	0.079	0.002
	NET	0.278	0.181	0.343	0.007	0.228	0.133	0.301	0.007	0.050	0.030	0.082	0.002
	JR	0.129	0.042	0.239	0.010	0.051	-0.046	0.178	0.285	0.078	0.040	0.133	0.002
	PA	0.211	0.134	0.311	0.003	0.211	0.134	0.311	0.003	—	—	—	—
Turnover intention	SE	0.379	0.319	0.430	0.005	—	—	—	—	0.379	0.319	0.430	0.005
	OM	0.332	0.254	0.410	0.004	—	—	—	—	0.332	0.254	0.410	0.004
	JD	0.138	0.034	0.239	0.011	0.117	0.009	0.215	0.034	0.021	0.004	0.052	0.013
	NET	0.222	0.133	0.317	0.002	0.196	0.096	0.287	0.004	0.027	0.008	0.053	0.015
	JR	0.350	0.247	0.441	0.004	0.307	0.195	0.411	0.004	0.042	0.013	0.094	0.013
	PA	0.114	0.029	0.212	0.020	0.114	0.029	0.212	0.020	—	—	—	—

Note: SE,Social environment risks; OM,Organizational management risks; JD,Job demand risks; NET,Negative event risks; JR,Job resource risks; PA,Psychological adjustment risks; PS,Psychological health status.
